# Complex realities: community engagement for a paediatric randomized controlled malaria vaccine trial in Kilifi, Kenya

**DOI:** 10.1186/1745-6215-15-65

**Published:** 2014-02-25

**Authors:** Vibian Angwenyi, Dorcas Kamuya, Dorothy Mwachiro, Betty Kalama, Vicki Marsh, Patricia Njuguna, Sassy Molyneux

**Affiliations:** 1 The Kenya Medical Research Institute (KEMRI)/Wellcome Trust Research Programme, P.O. Box, 230-80108, Kilifi, Kenya; 2 The Ethox Centre, Department of Public Health, University of Oxford, Old Road Campus, Headington, Oxford OX3 7LF, UK; 3 The Centre for Clinical Vaccinology and Tropical Medicine, Nuffield Department of Medicine, University of Oxford, Old Road Campus, Headington, Oxford OX3 7LF, UK

**Keywords:** Community engagement, Informed consent, Malaria, Randomized controlled vaccine trial, Sub-Saharan Africa

## Abstract

**Background:**

Community engagement (CE) is increasingly promoted for biomedical research conducted in resource poor settings for both intrinsic and instrumental purposes. Given the potential importance of CE, but also complexities and possibility of unexpected negative outcomes, there is need for more documentation of CE processes in practice. We share experiences of formal CE for a paediatric randomized controlled malaria vaccine trial conducted in three sites within Kilifi County, Kenya.

**Methods:**

Social scientists independent of the trial held in-depth individual interviews with trial researchers (n = 5), community leaders (n = 8) and parents (15 with enrolled children and 4 without); and group discussions with fieldworkers (n = 6) and facility staff (n = 2). We conducted a survey of participating households (n = 200) and observed over 150 CE activities.

**Results:**

The overall CE plan was similar across the three study sites, although less community-based information in site C. Majority perceived CE activities to clear pre-existing concerns and misconceptions; increase visibility, awareness of and trust in trial staff. Challenges included: some community leaders attempting to exert pressure on people to enrol; local wording in information sheets and consent forms feeding into serious anxieties about the trial; and concerns about reduced CE over time. Negative effects of these challenges were mitigated through changes to on-going CE activities, and final information sharing and consent being conducted individually by trained clinical staff. One year after enrolment, 31% (n = 62) of participants’ parents reported malaria prevention as the main aim of the activities their children were involved in, and 93% wanted their children to remain involved.

**Conclusion:**

The trial teams’ goals for CE were relatively clear from the outset. Other actors’ hopes and expectations (like higher allowances and future employment) were not openly discussed, but emerged over the course of engagements. Encouraging open discussion of all actors’ intentions and goals from the outset takes time, risks raising expectations that cannot be met, and is complex. However, doing so in future similar trials may allow successes here to be built upon, and some challenges minimized or avoided.

**Trial registration:**

ClinicalTrials.gov NCT00866619 (registration 19-Mar-2009).

## Background

Community engagement (CE) is increasingly promoted for biomedical research conducted in resource poor settings [1], for both intrinsic reasons (for example to show respect and trustworthiness) and instrumental reasons such as strengthening science through improving acceptability and interest in research, and strengthening ethical practice through improving consent processes [2,3].

While few would argue with these goals of CE, the complexity and the contested nature of all key elements of CE are becoming increasingly recognised [4,5]. With regards to ‘community’, it is unclear how this should be defined, given that individuals are always members of multiple communities, with membership shifting over time and space. In non-participatory health research, communities are often defined in relation to the nature of the research activity (for example does it involve a particular geographical area or illness group?) and the location of the research institution (for example is it based in a rural or urban setting, and is the population relatively mobile or stable over time?) [6,7]. Empirical studies have also suggested that for health research, the Ministry of Health (MoH) and other key health providers are often important key communities to include [8]. Even where there is clarity on which key communities to engage with, how representatives of those communities should be selected, and how researchers should work with those communities to ensure that their contribution is recognised but their independence from researchers is maintained, is far from straightforward [6]. In terms of ‘engagement’, definitions can vary from many different formal and informal communication activities, through to more specific interventions such as deliberate employment of local community members by research organisations as field staff, and provision of services such as health care [9]. In an international meeting on consent and CE, participants reached a consensus to define CE primarily as communication activities, with the provision of health and other services and care considered as crucial activities (often informed by CE) but conceptually separate from CE. It was agreed that the provision of health and other services and care could instead be considered as part of research design (including ancillary care) and benefit sharing. Even where CE is considered as primarily about communication, engagement activities can cover a wide range of interactions including those aimed at consulting with community members about what should be done in studies, through those aimed at sensitising community members about research or specific studies, to activities aimed at ensuring communities receive feedback of individual test results or overall study findings [8,10]. These activities are often part of formally organised communication strategies and activities of research institutions. However, research staff collaborators and colleagues in Ministries of Health also have informal interactions about research and researchers in the multiple communities that they are members of. This web of informal communication and interaction can play a key role in community members’ perceptions of and relationships with individual research staff and the institutions they belong to [9,11-14].

Formal engagement activities can take place at any stage of research, although it is recognised that the amount of engagement often reduces over time as studies progress, with some arguing that feedback of study findings at the end of research is often given inadequate attention [15]. Engagement can be through structures that exist independently of research such as chiefs, elders, and women’s groups, or through structures specifically established by research organisations such as various forms of Community Advisory Boards or Groups (CABs or CAGs) [6]. The importance of ensuring that traditional decision-making structures such as chiefs, elders and health managers are engaged with has been highlighted in empirical studies [1,16,17]. However, also highlighted in the literature has been the importance of ensuring representation of those with less authoritative positions in communities; of ensuring that both ‘typical’ members of communities and the most vulnerable and often least visible and vocal groups are heard [9]. In practice, communication activities often involves a variety of different structures and groups, with activities of varying sizes potentially being conducted in many different settings, including homes, communities, health facilities and research centres. Where meetings are held and how large and regular they are, has implications for who is willing and able to attend, how much information can realistically be covered in what depth, how much interaction and discussion is possible, and how free participants feel to raise their views and concerns [6,18].

A fundamental challenge in practice for formal CE strategies is that researchers’ goals of CE are often implied rather than clearly articulated [5,8]. This is an important challenge because many CE initiatives have diverse goals, which may be in tension with each other, and because all CE has the potential to have a negative impact, at the very least through taking up people’s time, but also through unintended perverse outcomes such as making some individuals feel obliged to take part in research through peer pressure, or raising expectations that cannot be met [1,6,16,19,20]. Another reason that greater clarity in goals is important is that there is clearly a limit to what ethical issues CE itself can resolve in research, including those related to historical and background injustices and inequities, and unfair distribution of risks and benefits in research [5]. Recognition of the limits of CE, as CE is defined here, is important in highlighting the need for broadening the scope of CE beyond communication activities, and the feasibility of undertaking these; for incorporating other initiatives and thinking to strengthen ethical research practice. Beyond clarity in CE goals, other fundamental issues in practice in CE include taking into consideration who drives the CE agenda, how power relations feature in community-researcher interactions, and how research findings are shared with policy-makers and implementers in a position to influence policy and practice in the longer term [4].

Relatively few recent studies have examined CE processes in practice. Given the potential importance of CE, but also the complexity and potential for perverse outcomes such as those listed above, there is a recognised need for more documentation of micro-processes as they unfold on the ground. Such research needs to go far beyond documenting simple consent rates: participation in research is only one possible goal of CE, and achieving this goal can compete with other goals such as strengthening understanding of the trial and its’ implications for individual participants. Moreover, participation rates are also influenced by many factors beyond CE [8].

In this paper we share on-going experiences of formal CE for a paediatric randomized controlled malaria vaccine trial conducted in three different sites in one geographical area of Kilifi County on the Kenyan Coast. The trial was conducted in three local health facilities, and involved healthy infants and children. Although the trial is on-going, we draw on data collected between 2009 and 2011 from a large mixed method study running concurrently with the trial. Following an overview of the study setting, trial, and qualitative study methods, we describe the processes followed in CE, how these changed over time, the goals of involving different community actors in CE processes from the trial team’s perspective, and trial staff and community members’ perceptions of the successes and challenges of the approaches followed. We then discuss the levels of understanding of the trial among participants’ parents, measured one year after consenting. Finally, we draw on our data and the wider literature to show that the complexity of CE in our setting was exacerbated by the very different realities, goals and interests that the various actors brought to the activities and interactions, including diverse and multi-layered practices of power. The implications for future CE activities and how to evaluate these are discussed.

### Study context

#### Study setting and CE activities

The paediatric randomized controlled malaria vaccine trial began in 2009 and is on-going (ClinicalTrials.gov; NCT00866619); see also, [21]. It is being conducted by the Kenya Medical Research Institute/Wellcome Trust Research Programme (KEMRI/WTRP, or KWTRP) in several administrative divisions within Kilifi County, on the Kenyan Coast. Kilifi County has low literacy levels, high poverty rates, and farming is the main occupation [22]. KWTRP has a long history of multi-disciplinary research in Kilifi County, including empirical studies on consent and CE.

All studies conducted by KWTRP in Kilifi County are supported by the Community Liaison Group (CLG); a group of six facilitators, four senior fieldworkers (FWs) and a Community Liaison Manager, responsible for ensuring that mutual understanding is built, and that there are strong, honest and supportive relations between staff and local residents [23]. The CLG develop and implement annual plans for CE activities in the programme, supported by three senior researchers with an interest in research ethics. CLG activities can be broadly divided into programme-wide CE, and project-specific engagement activities. Programme-wide CE activities focus on the 260,000 people living within the Kilifi Health and Demographic Surveillance System (KDHSS) area [22], and include: information sharing on what research is and how participants’ rights are protected in research, and on new studies; consultation with community representatives (chiefs, leaders, and typical community members) on planned or on-going research or research policy; and feedback of research findings. Programme guidelines have been developed to support study teams to design appropriate CE plans for each study. To support study-specific CE plans, CLG members and trial representatives sit on a Community Advisory for Study Teams (CAST) set up for each study that requires CE. Through this mechanism, CLG members advise on activities with the aim of ensuring that issues raised through interactions with community members are discussed with key stakeholders at the Programme and externally where appropriate.

**Figure 1:**
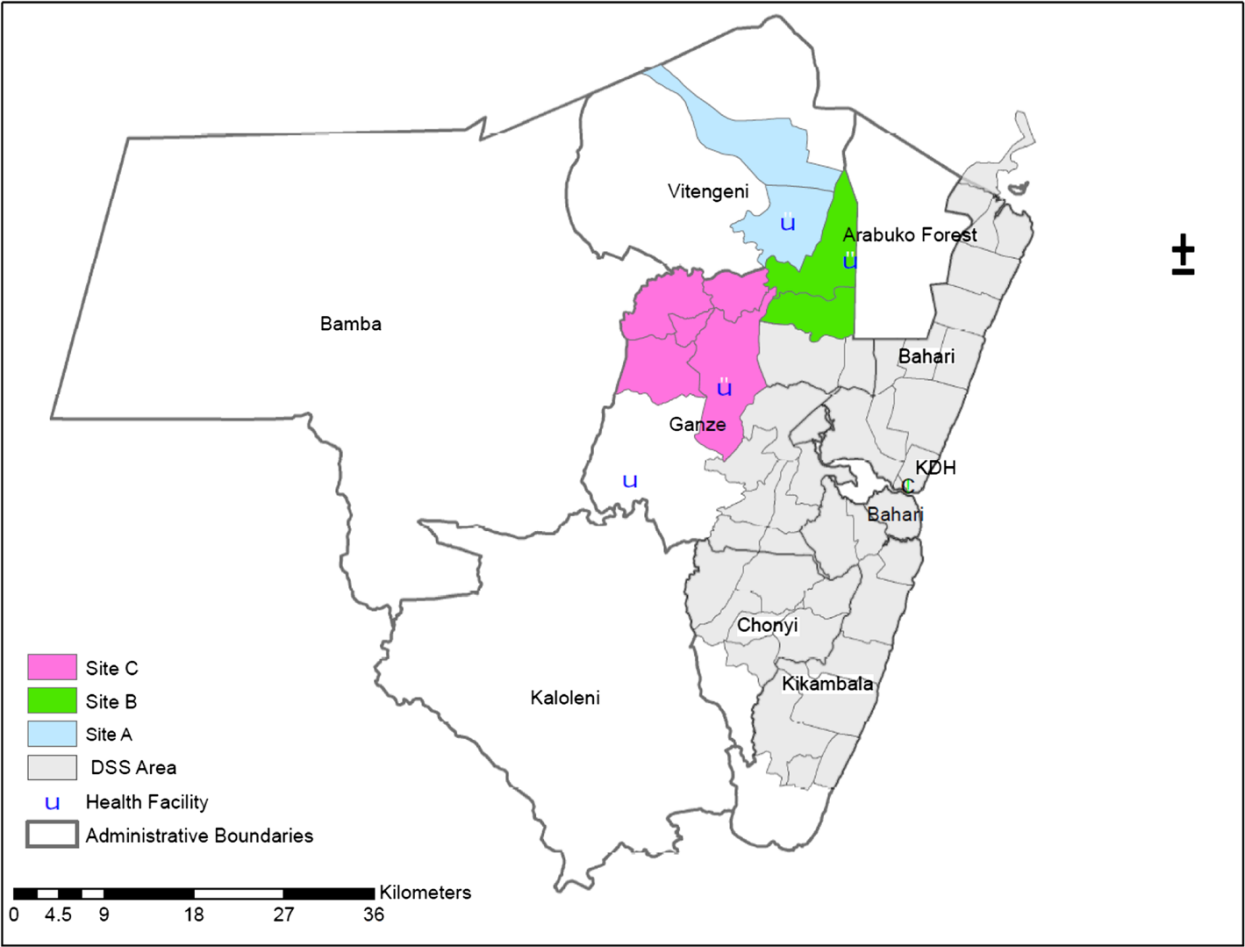
Map of trial sites.

The CE discussed in this paper was for a trial being conducted outside the KHDSS, in two locations bordering the KHDSS but further inland, involving three rural health facilities referred to here as sites A, B and C respectively (see also Figure 1). The locations where the trial is being conducted had very little direct involvement with the research organisation prior to this trial, and there were previously no programme-wide CE activities being regularly conducted. A CAST group was therefore set up for this trial, including CLG members (facilitators and a senior fieldworker) and trial team staff (a senior trial researcher and manager) to advise on the design and implementation of CE activities. The CE plan developed by the CAST group drew upon previous findings and experiences of a similar trial conducted in other parts of Kilifi District [8]. The overall aims outlined in the strategy were to:

• Consult with community representatives and other key stakeholders on how to work in facilities and communities, and who to engage with and how;

• Create awareness of KEMRI and the trial in these new sites;

• Gather community leaders’ opinions of the study;

• Build trust with key community leaders and representatives; and

• Provide a forum for discussing and addressing issues arising from participants and community representatives.

#### Trial design

The trial is described in detail elsewhere [21]. In summary, 904 children aged 5 to 17 months and 6 to 12 weeks at enrolment were recruited from villages surrounding three rural local health facilities in Vitengeni and Ganze district. The trial aimed to document the vaccines’ safety, immunogenicity and efficacy against febrile malaria in children through a double-blind randomized controlled design; the primary outcome is clinical malaria episodes. For participating children the trial involved: an initial health check; randomization into one of three arms, that is malaria vaccine only, malaria vaccine and comparator, or comparator vaccine only; four vaccination visits to the health facility; and monthly home visits by a FW for four years. The trial vaccines were co-administered with routine childhood vaccines and given at months 1, 2, 3 and a booster dose administered at month 18. Participants’ parents were encouraged to report any health concerns to the study team as soon as possible. Study benefits for participants included free treatment for any illnesses, payment of transport costs for any visits to the health facility, and an ability to communicate with locally resident trial staff (fieldworkers; FW) 24 hours a day. FWs were in turn able to phone medically qualified staff to facilitate referrals, where necessary. Refreshments were provided during study-organised health facility visits.

## Methods

### Study design

The data from this paper were collected by social scientists with strong links with, but who were not part of, the KWTRP Community Liaison Group, or the trial team. The study design was a mixed methodology descriptive approach combining individual and group in-depth interviews and surveys, observations and document reviews. In addition to each method making a unique contribution to understanding complex realities of CE for this trial, using multiple methods allowed us to continue to evolve our research tools and understanding. For example observations fed into interview tools and vice versa, and the survey questions drew upon in-depth interview information and tested commonness of points made. In this paper we present data collected between 2009 and 2011; a period of intense activity for the trial.

### Non-participant and participant observation

We conducted both participant and non-participant observation. We participated in the trial’s CE CAST meetings, and observed consent and CE activities in the community and trial facilities. We observed community meetings in villages (n = 42 meetings), accompanied FWs and community facilitators on visits to potential participants’ homes (n = 87 home visits), and attended 31 facility-based information-giving sessions. Semi-structured observation tools captured verbal and non-verbal information, including who attended and participated in activities, what information was covered, what questions were asked, and how questions and concerns were handled.

### In-depth interviews (individuals and groups)

Interviews aimed at exploring people’s experiences and perceptions of the trial’s informed consent process and CE activities across the three trial sites. Interviewees were purposively selected to maximise diversity of experience, taking into account for example, role in the trial, enrolment status, gender and location. Individual in-depth interviews (IDIs) were held with (see Table 1): trial researchers (n = 5); community leaders (n = 8) including chiefs, village elders, health facility committee representatives and community health workers (CHWs); and parents (15 with enrolled children and 4 without). Group discussions were also held with health facility staff (n = 2 groups; 5 staff in total) and trial FWs from the three sites (n = 6 groups; 20 FWs in total with most interviewed twice). Interviews covered the following topics: how communities were involved and informed about the trial, and what influenced their decision-making about joining or refusing; their understanding, views and experiences of the trial and of CE activities; and their own and others’ ability to communicate concerns to the trial team. We also sought recommendations for future similar trials.

**Table 1:** Respondents and methods used (qualitative data)

**Category of respondents**	**Data type**	**n**	**Number of respondents**
Community members	IDIs with parents of children enrolled in the trial	15	15
	IDIs with parents of children not enrolled in the trial	4	4
	Household semi-structured survey with parents of children in the trial	200	200
Trial staff	Trial staff IDIs	5	5
	Fieldworkers FGDs	6	20 fieldworkers
Health workers and leaders	Health facility staff group interviews	2	5
	Community leaders IDIs	8	8

FGD, focus group discussion; IDIs, in-depth interview.

### Semi-structured household survey

A household survey, administered one year after recruitment, aimed at exploring similar topics to in-depth interviews among many parents/guardians of children enrolled in the trial, to have some sense of commonness of findings across participants. We were particularly interested in exploring whether levels of understanding among parents/guardians of trial participant differed by site, and particularly sites A and C (the start and end) on the basis of initial qualitative data. Survey respondents were randomly selected from the trial database of all participants, proportional to the total number of trial participants per site. In total, 200 participants were surveyed; 67 from site A, 33 from site B and 100 from site C. Of the 200 sampled households for this study, only one had to be replaced since we could not find them after three consecutive visits. Demographic characteristics of survey respondents are highlighted in Table 2.

**Table 2:** Characteristics of survey interviewees (Household survey)

	**Site A n = 67 (%)**	**Site B n = 33 (%)**	**Site C n = 100 (%)**	**Total n = 200 (%)**
*Sex*				
Male	6 (9.0)	3 (9.1)	5 (5.0)	14 (7.0)
Female	61 (91.0)	30 (90.9)	95 (95.0)	186 (93.0)
*Age, years*				
16 to 24	15 (22.4)	7 (21.2)	24 (24.0)	46 (23.0)
25 to 44	50 (74.6)	25 (75.8)	72 (72.0)	147 (73.5)
45 and above	2 (3.0)	1 (3.0)	4 (4.0)	7 (3.5)
*Religion*				
Christian	36 (53.7)	19 (57.6)	46 (46.0)	101 (50.5)
Muslim	6 (9.0)	0	19 (19.0)	25 (12.5)
None/traditional	25 (37.3)	14 (42.4)	35 (35.0)	74 (37.0)
*Highest level of education*				
None	23 (34.3)	9 (27.3)	39 (39.0)	71 (35.5)
Some primary education	34 (50.8)	23 (69.7)	54 (54.0)	111 (55.5)
Some secondary education	5 (7.5)	1 (3.0)	3 (3.0)	9 (4.5)
Adult education	4 (6.0)	0	3 (3.0)	7 (3.5)
Tertiary and above	1 (1.5)	0	1 (1.0)	2 (1.0)

### Document reviews

To supplement the above activities, we reviewed the KWTRP CE guidelines, the trial’s CE plan and minutes from all CE-related meetings.

### Data management and analysis

Interviews, observations and the household survey were conducted primarily by the first and second authors (VA and DK) together with four trained senior (non-trial) FWs who speak the local dialect. Community interviews were conducted in Kiswahili or Kigiriama as preferred by the interviewees.

Qualitative interviews were audio taped, transcribed and translated into English (where necessary), and coded and analysed using a framework approach [24] in NVivo 8 (QSR International, Melbourne, Australia). VA and SM developed the initial coding framework based on the study objectives and a review of a sub-set of the data, with initial themes including: CE goals (at the outset of the trial, over time, and at the end of the trial); understanding of and perceptions of the KWTRP, the trial, and CE activities; and unexpected or unintended outcomes of CE. The framework was then periodically amended to incorporate emerging sub-themes such as the contrasting goals of different key actors involved in CE. All coded data were exported into charts in MS Word 2007 (Microsoft, Washington, USA) to explore the diversity of viewpoints by categories of actors and trial sites and emerging patterns. For observational data, detailed notes were transformed into summaries based in part on the interview themes. Survey data were double entered in Visual FoxPro v9 (Microsoft, Washington, USA) and later exported to STATA v11 (StataCorp, Texas, USA) to conduct simple frequencies, Pearson’s chi-square test of association (where necessary) and cross-tabulations of key information by trial site (based on qualitative data indicating its importance).

### Ethical considerations

Ethical approval for the trial itself was obtained from institutional review committees and the National/KEMRI Ethical Review Committee before the trial began (Protocol Number: SSC1445). Approval for this mixed methodology sub-study was obtained later and separately (Protocol Number: SSC 1541). All sub-study data were anonymized, and stored in password protected computers and in lockable cabinets with personal identifiers removed. Consent was obtained from interviewees. Feedback meetings among the social science team, and with key members of the trial team, were held regularly to ensure that emerging issues were shared and acted upon by the trial team.

## Results

Following an overview of the trial CE strategy and of the variations by facility, or site, we describe in turn: the views of the early activities leading to trial recruitment (community consultation, entry and sensitisation); the levels of trial understanding among participants’ parents (one key aim of early CE activities); and continuous engagement and feedback of findings. As observed elsewhere, much of the CE focussed on the early activities, which included strengths and challenges that we discuss related to: involvement of existing community gatekeepers and leaders; aligning and embedding activities in existing activities and structures; and the terms used in information sheets and consent forms.

### CE strategy and variations by site

The formal and basic CE activities which took place over the trial’s first few years are summarised in Table 3. Through observations and interviews, we noted particular emphasis at the outset on consultation and planning with key stakeholders, including the Provincial Administration (district officers, chiefs and village elders) and the MoH (district health managers, the local health facility staff and CHWs.

**Table 3:** **Summary of community engagement activities**^**1**^

**Period**	**Activity**	**Who involved**
Month 1	Consultation and sensitisation of Kilifi District stakeholders	1. MoH structure: District Medical Officer of Health and District Health Management Team at Kilifi District Hospital. All health facility in-charges working in Kilifi District
		2. Provincial administration structures: District Commissioner, Senior District Officer, all chiefs working in Kilifi District
Months 2 to 6	Community entry and sensitisation of stakeholders in sites A, B and C respectively	1. MoH Structure: Dispensary health committees, dispensary staff (facility in-charges, nurses, public health officers, community health extension workers), and community health workers (CHWs)
		2. Local administration: District officers, local (assistant) chiefs and village elders
		3. Others: primary school head teachers, religious leaders, a local District Stakeholders Forum
Months 8 to 13	Identification and recruitment of 5 to 17 month-old children (n = 600)	CHWs and fieldworkers
Months 15 to 27	Identification and recruitment of 6 to 12 week-old children (n = 304)	CHWs and fieldworkers
From month 8 to date	Follow-up of research participants	Fieldworkers
	Continuous feedback to and from community	Fieldworkers and other key gatekeepers
	Feedback of results	Involves all of the above, for example, preliminary study results disseminated

^1^Source: Angwenyi V, Kamuya D, Mwachiro D, Marsh V, Njuguna P, Molyneux S: **Working with community health workers as ‘volunteers’ in a vaccine trial: practical and ethical experiences and implications**. *Dev World Bioeth* 2013, **13**:38**–**47.

Sensitisation and recruitment activities were then implemented in site A, followed by site B and then site C. In each site, initial consultations with community leaders were followed by sensitisation meetings for the general community, but particularly targeting families with eligible children. Meetings were organised by trial staff in liaison with community leaders, with key messages developed by the CAST group. Sensitisation activities were followed by consent and recruitment activities, and over the course of the trial, field staff and community leaders were generally considered by the research team as the channel for community members to voice any concerns. As is often the case [5], consent processes for this trial were embedded within the wider CE activities, but considered as a separate, more specific and individualised activity. From the outset, feeding back of individual and research findings at the end of the trial was considered essential [15].

Although the overall community sensitisation plan for key stakeholders (chiefs, village elders, CHWs, health facility staff and health committees) was the same across sites, some changes were reported and observed over time. These changes are summarised in Table 4, but two illustrative examples are described here. Firstly, there were changes in the extent of involvement of key community stakeholders, and the amount of community based information sharing and sensitisation. For instance, CHWs were increasingly involved in giving general information about the trial to potential participants in the community, but over time there was generally less community based information sharing; that is there were fewer household visits and fewer large public meetings in sites B, and especially C, than in A.

**Table 4:** Summary of changes in community engagement by site and over time

**Study site**	**Site A**	**Site B**	**Site C**
Prior exposure to KEMRI	Less	Less	More
Perceived ease on recruitment/amount of CE activities	Harder/more CE	Easier/moderate CE	Easier/less CE
CE and consent process changes (across sites)	• Provider of initial trial information: more by CHWs but less detailed		
	• Provider of detailed study information: more by experienced facilitators and clinicians		
	• Disclosure setting of initial trial information*:* more in community than in homes		
	• Consent setting: more in facilities than in homes		
	• Time consent is sought: closer to time of recruitment; less time for intra and inter family consultation and discussion		
	• Administration of consent: less access in advance to consent forms with ‘scary wording’		

CE, community engagement; CHW, community health worker.

Trial staff attributed these changes to less need for large scale community based information over time because of fewer concerns about the purpose, amount and use of blood taken, and about trial safety. In site A, these concerns reportedly led to numerous questions from community members, and apparently fuelled rumours about KWTRP. Commonly reported rumours were KWTRP being ‘devil worshippers’ and children being likely to die on receipt of the vaccine. Such rumours not surprisingly led to further questions and concerns, and to low recruitment rates, and to more community meetings than had initially been planned, to supplement household follow-up visits:

…there should be barazas [public meetings], so that those who are not involved they will be able to ask and get answers to their questions. But this [research] didn’t come that way. Initially after they informed leaders, village elders and CHWs, they went straight to meet people who had [eligible] children. So this is what brought problems they forgot that there are those others whom we call ‘wajuaji’ [referring to knowledgeable people/influential figures - no direct translation], who were left out … [yet] they are listened to in the community (IDI01_male, community leader, site A).

… If they could have started with meetings [in a central place] and then gone around to people’s homes then they would have succeeded. It’s like climbing a tree from the top to the bottom, when everybody knows you should start from the bottom and climb up’ (IDI07_male, parent, non-consenter, site A).

Although more public meetings or *barazas* were organised to inform a wide range of community members, challenges with such meetings is the absence of many influential figures, especially fathers of young children, and inability to cover detailed information and have one-to-one discussions. Thus, home visits remained a key sensitisation strategy in all three sites:

… a lot of them [women] have to seek consent from their husbands, and therefore if the husband says [in a household visit] ‘oh yeah you go listen and join the study’ , then they are more likely to … [since] they already have permission if I may say so. But if you just speak to them [women] in a baraza [public meeting] and then they go explain to their husbands, the response is not so good (IDI04_female, trial staff).

A second example of a change over time is regarding who administers consent and where. Initially, consent information was provided in potential participants’ homes by FWs employed from the local community. In household interactions and CAST discussions, it was noted however that newly trained FWs could not always address emerging and technical questions about the trial, and that parents might feel pressurised to consent when visited in their homes. FWs’ roles were therefore limited to assisting in general information in the community. Parents interested in participating were invited to visit health facilities for more detailed study information provided by experienced community facilitators and trial clinicians. Group information was followed by individual consent processes led by clinicians who speak the local dialect.

### Community consultation, entry and sensitisation

Overall, trial staff, community leaders and participating households reported that CE activities and interactions helped clear pre-existing concerns and misconceptions about KEMRI’s work; raised awareness; built trust; and increased the visibility of KEMRI staff initially seen as ‘outsiders’.

It was very, very hard in fact at the initial stages … KEMRI was being associated with devils, and that was the story in the community then, but I commend the entry point. Before they started this whole project there was very, very good interaction between you people [KEMRI staff], and how you entered these communities through DHCs [dispensary health committees], chiefs, and barazas [public meetings]. It helped a lot. I think that’s why after a short time community members accepted because now I don’t hear any problems of maybe community members complaining … (Group interview2_male, health facility staff, site B).

… the understanding of KEMRI here is much, much more positive than it was … because of the approach that they [KEMRI] used. The concern of the local people particularly about this research was mistrust, mistrust of KEMRI as a body [institution] … it [the approach used] has helped give a positive image of KEMRI we can really trust (IDI02_male, community leader, site A).

The overall importance of the initial CE, and the strategies used, was supported by participating households in their responses to the survey. Over 95% of survey respondents recommended that future similar trials should inform each of chiefs, village elders, parents of eligible children and health facility staff in advance of trials, and 91% mentioned CHWs (Table 5).

**Table 5:** Sources of information prior to and post joining the trial (Household survey)

	**Prior to joining the trial n (%)**	**Post joining the trial n (%)**	**Perceptions on who to approach before a trial starts in future similar studies n (%)**
FWs at home	138 (69.6)	120 (74.5)	N/A
FWs elsewhere	49 (27.1)	24 (50.0)	N/A
Dr./KEMRI staff (facility)	138 (71.3)	78 (48.7)	N/A
Dr./KEMRI staff (home/village)	87 (45.8)	25 (34.7)	N/A
Friends/neighbours	93 (46.7)	30 (35.3)	132 (72.9)
Health facility staff	49 (24.8)	24 (45.3)	171 (96.1)
CHWs	103 (52.6)	24 (23.3)	169 (90.9)
Village elders	104 (52.8)	22 (23.9)	194 (99.0)
Chiefs (sub-chiefs)	97 (49.0)	26 (30.2)	196 (99.5)
Religious leaders	19 (9.6)	8 (40.0)	140 (76.5)
Traditional healers	4 (2.1)	3 (33.3)	53 (29.1)
Parents with eligible children	N/A	N/A	184 (96.3)
Others (specify)	31 (19.0)	9 (25.0)	41 (24.1)

### Involvement of existing community gatekeepers and leaders

Most staff, parents and community leaders interviewed reported that it was essential to incorporate pre-existing decision-making structures such as district officers, chiefs and elders into CE strategies. These individuals reportedly helped build trusting relationships between researchers and community members because they are well recognised, trusted and respected:

You know when something new is starting, it always has problems. Like the leaders; they were ring leaders explaining that this [research] had no problems. Communities really trust their area leaders rather than just anybody coming from nowhere to go and explain to them … (FGD02_male fieldworker, site C; researchers’ emphasis).

Some community leaders reportedly went ‘an extra mile’ to support the trial and minimise concerns through spontaneously organising their own sensitisation meetings, and even enrolling their own children:

… someone like the assistant chief … recruited his children in the study and that was like a good mirror to the community. So he would even say ‘I have enrolled my children in the study and since I joined there have been no effects. So you should not spread propaganda that KEMRI is bad, no!’ So this to me and even to the community was something good (FGD02_male, fieldworker, site C).

We called all pastors here, CBO [Community Based Organisation] leaders … and then we brought in representatives from health facilities and we had a meeting here [at the office]. So ours basically was to try and seek how the combined efforts can help us inform the general community and educate them to know that KEMRI does this and this (IDI02_male, community leader, site A).

There were also some challenges with involving community gatekeepers and leaders. There were numerous requests for assistance, for example for lifts from KEMRI vehicles, for allowances, for construction or decoration of offices, and for bicycles or future employment. Moreover, during recruitment difficulties, some chiefs and leaders apparently attempted to ‘exert pressure’ on people to enrol, through demanding from elders why recruitment challenges are being faced (site A), threatening to withdraw people from a local food for work development project (site A), and saying they would arrest rumour mongers (site C). Some of these incidents clearly presented potential threats to the voluntary nature of research participation and had to be addressed by the trial team through subsequent information giving sessions.

… village elders tended to be coercive sometimes … you could go with them [to homes] and you know that … [research] participation is voluntary. But then for a village elder, because he wants the [research] agenda fulfilled then he says, ‘we want everybody who has an eligible child to join the study, or else we will make sure you are removed even from other government projects that are brought here’. So in such a place you [fieldworker] have to come back again and try to explain that this [research] is not a must, it’s voluntary (FGD01_male, fieldworker, site A).

In some cases, unexpected and unintended initiatives by gatekeepers were appreciated by community members, illustrating the complex potential role that such actors can play in information sharing and recruitment:

… we went to the meeting and the assistant chief said he doesn’t want to hear any rumours from anybody. He said he was an assistant chief and he had a child who was in the KEMRI study. He told people if they won’t enrol in the study they should just keep quiet and anybody who will be heard spreading rumours will be arrested … After the assistant chief called the meeting, rumours stopped and for us who had already joined, we felt a bit better because rumours had stopped (IDI11_female parent, late consenter, site C).

Parents recognised the key role of community leaders in assisting with information giving and addressing community concerns. However, for decisions on whether or not to involve their child in the trial, those most often mentioned as being consulted by parents were spouses and close family members (55.0%; n = 110). Almost all parents (91.5%, n = 183) reported the final decision to enrol was theirs and voluntary.

### Aligning and embedding CE in existing structures and activities

Consultations with community gatekeepers were described by researchers as helping to identify additional ways of communicating about the research. For instance, MoH officials strongly promoted from the outset using existing structures like CHWs rather than creating parallel community representative groups such as community advisory boards, or KEMRI community representatives; see also, [10]. Aligning CE activities for the trial with those already planned by key community gatekeepers was considered by most interviewees as an appropriate approach to reach out to as many people as possible with study information (Table 6).

… there were those [meetings] which community [leaders] themselves had already organised according to their calendar. We could get a bigger crowd [and] then ask for a slot [within that larger meeting] to sensitise the community about the study. For the meetings which we organised [ourselves], initially they were a bit difficult. Sometimes you organise for a meeting and when we go for that meeting, very few people turn up (IDI05_ male, trial staff).

**Table 6:** Key actors (and those suggested) for community engagement activities (observation and interviews data)

**Actors - who are they**	**Study team goals for engagement**	**Un-anticipated activities**	**Revealed personal goals for involvement in engagement**
Administrative authority (includes District Officers, Chiefs and Village elders)	1. Consultation and entry. For example:	1. Conducted additional sensitisation activities in the absence of KEMRI staff and warn people to avoid politicizing the research process in community *barazas* (public meetings)	1. Contribute to success of the trial**,** defined primarily as achievement of sample size and general smooth running
	• Seek support and permission to carry out the trial	2. Asked participants to speak about their experiences in public meetings	2. Community health benefits, for example, construction/ expansion of HF and improved quality of care (ambulatory services, skilled staff, and so on)
	• Gather opinions on who should be targeted with study information and identify other opinion leaders	3. Intervened during low recruitment to emphasize others to join the trial, including through demanding from leaders why recruitment challenges are being faced (site A), and threatening to withdraw people from food for work project (site A), and to arrest rumour mongers (site C).	3. Personal or professional gains, for example, lifts from KEMRI vehicles, allowances when engaged in activities, increased status/recognition through affiliations with KEMRI/staff, assistance in constructing offices, and being seen as successful, that is, through introducing a (quasi) government project
	2. Sensitisation and mobilisation. For example:	4. Made many requests for personal or professional gains, for self, or others.	
	• Create awareness of KEMRI and understanding of trial activities in their jurisdiction		
	• Assist with meetings for other opinion leaders and community members through *barazas* (public meetings)		
	3. On-going communication. For example:		
	• Be aware of trial and procedures if contacted by concerned community members		
	• Forward community issues to study team where necessary		
Community organisations/forums (for example, a local district stakeholders’ forum)	1. Sensitisation and mobilisation. For example:	1. Assisted in giving simplified information at large meetings. Assisted in clearing community concerns about KEMRI’s work	1. Gains to the organisations that they are part of - for example membership fee to the organisation
	• Create awareness of KEMRI and understanding of trial activities	2. Made some requests to KEMRI for the organisations that they represent	2. Protection of the community against risks and concerns
	• Encourage interested community members to hear more about the trial		
Fieldworkers (these are local community members employed to undertake certain study procedures)	1. Benefit community and build trust through employing members using transparent mechanisms	1. Communicating constantly with community members through their roles and in their personal lives as they live in the community, including through handling numerous questions	1. Meet recruitment targets to maintain jobs
	2. FWs conduct their formal study roles which include sensitisation and mobilisation, constant feedback to study team, and feedback of individual and study findings	2. Emphasise study benefits in all interactions	2. Build relationships that support recruitment

HF: health facility.

However, contrasting advice from different community gatekeepers presented the trial team with dilemmas. Thus for example, some community leaders suggested that traditional healers should receive targeted sensitisation, whereas facility staff and MoH managers were adamant that traditional healers should not be included because they are not officially recognised by the MoH. In this case, a compromise was reached: there were efforts to include healers in wider community meetings, but they were excluded as a specific CE channel. Another example was that a location-level stakeholder group wanted KEMRI to be a member and attend their meetings. However, membership required payment, which raised concerns that this would potentially lead to payment requests from numerous other groups, or might be interpreted as ‘buying’ local support. In this case, after extensive consultation, KWTRP (as opposed to the trial team specifically) did join and pay the nominal membership fee; none of the anticipated problems have yet been reported.

In terms of involving CHWs, we have discussed the complexities of this in detail in another paper [10]. Briefly, CHWs were initially engaged as an important network *to be informed* about the trial. However over time, and in response to community advice, they became involved in trial information sharing and identifying potential participants; thereby taking on roles that overlapped with those of employed FWs. While CHWs’ involvement was generally perceived as positive and appreciated, there were challenges in their relations with FWs and with other community members, partly related to different levels of remuneration.

### Concerns associated with approved study information sheets

An issue recognised and discussed often in public meetings and in homes particularly at the outset of the study, and linked to pre-existing concerns and rumours about KEMRI being ‘devil worshipers’, was some of the local wording in information sheets and consent forms for compensation and randomisation. Particularly problematic was the translation of ‘randomisation’ into ‘*pata potea*’; (which translates to win or lose) a local dice game, with animals drawn on the dice instead of numbers. Rather than being interpreted as being assigned to the experimental vaccine (win) or control (lose), the explanation was understood to mean the possibility of losing a child. This also fed into perceptions that the experimental vaccine was already known to work.

Related to concerns about *pata potea*, some participants also interpreted compensation for adverse events to imply a high possibility of death. A FW summarises the concerns in the community:

… there was a form [informed consent] which was written that if the child dies you will be given ‘fidia’ [compensation] … when my wife and I heard that there is ‘fidia’ [compensation], she said I don’t want (laughter) … if I give them my child and the child dies, then they give money, that will not bring my child back. We had to explain to her until she understood. And in fact, some refused at that stage when they heard of KEMRI giving ‘fidia’ [compensation], they said ‘we don’t want our children in it. You want to take our child and give that child to the devils’ …’ (FGD04_male, fieldworker, site A).

The *confidentiality* clause in the informed consent form (ICF) also appeared to cause apprehension amongst some participants; increasing rumours and concerns about why KWTRP activities should be shrouded in secrecy. They wondered why confidentiality was important when most people in their community knew of their research participation.

There is this part of confidentiality, the locking of documents in cupboards; sometimes when you explain that to your participants they wonder about it … it’s like something will happen in the future which will surprise them … [that] information that is kept in cupboards and in the computers, why does it have to be secret? (FGD05_male, fieldworker, site B).

### Levels of trial understanding among participants’ parents

The household survey included questions on understanding of the trial, and on processes and voluntariness in decision-making. It should be reiterated however that these questions were asked one year after initial recruitment and informed consent (Table 7).

**Table 7:** Consent processes and understanding of the trial (Household survey)

	**Site A**	**Site B**	**Site C**	**Total**
	**n = 67 (%)**	**n = 33 (%)**	**n = 100 (%)**	**n = 200 (%)**
*Main purpose of the trial*				
Provide treatment/help to participating children	32 (47.8)	18 (54.5)	71 (71.0)	121 (60.5)
Learn about potential for preventing malaria	27 (40.3)	12 (36.4)	23 (23.0)	62 (31.0)
DK	3 (4.5)	1 (3.0)	3 (3.0)	7 (3.5)
Other activities	2 (3.0)	0	2 (2.0)	4 (2.0)
Missing/data not clear to code	3(4.5)	2 (6.1)	1 (1.0)	6 (3.0)
*Did all children in the trial get a malaria vaccine?*				
Yes	38 (56.7)	19 (57.6)	63 (63.0)	120 (60.0)
No	12 (17.9)	6 (18.2)	11 (11.0)	29 (14.5)
DK	17 (25.4)	8 (24.2)	24 (24.0)	49 (24.5)
Missing data	0	0	2 (2.0)	2 (1.0)
*Do you think children who got trial vaccines are still able to get malaria?*				
Yes	10 (14.9)	5 (15.2)	15 (15.0)	30 (15.0)
No	32 (47.8)	17 (51.5)	56 (56.0)	105 (52.5)
DK	25 (37.3)	11 (33.3)	28 (28.0)	64 (32.0)
Missing data	0 (0)	0	1 (1.0)	1 (0.5)
*Would consider participating in future similar trial?*				
Agree	45 (67.2)	20 (60.6)	80 (80.0)	145 (72.5)
Not agree	2 (3.0)	0	0	2 (1.0)
Indifferent/DK^a^	20 (29.9)	13 (39.4)	20 (20.0)	53 (26.5)
*Content with decision to enrol your child in the trial?*				
Yes	61 (91.0)	31 (93.9)	94 (94.0)	186 (93.0)
No	4 (6.0)	0	2 (2.0)	6 (3.0)
Indifferent	2 (3.0)	2 (6.1)	4 (4.0)	8 (4.0)
*Most important reasons for enrolling in trial (top five)*				
Access to free treatment	18 (26.9)	10 (30.3)	35 (35.0)	63 (31.5)
Access to better and quality services	9 (13.4)	6 (18.2)	25 (25.0)	40 (20.0)
Vaccine beneficial	12 (17.9)	5 (15.2)	13 (13.0)	30 (15.0)
Detailed explanation of the study	5 (7.5)	3 (9.1)	7 (7.0)	15 (7.5)
Previous experience/interaction with KEMRI	3 (4.5)	4 (12.1)	6 (6.0)	13 (6.5)

^**a**^Here most people reported they would first want to hear more about what the trial would involve at the point of information giving before making their decision.

DK; do not know.

Survey results indicate fairly low levels of understanding one year post recruitment. For instance although almost all parents were aware that their child was receiving a vaccine (96%, n = 192), only 31% (n = 62) reported that the main purpose of the activity was to learn about the vaccine’s potential to prevent malaria; 14.5% (n = 29) of parents reported that not all study children received a malaria vaccine and 15% (n = 30) reported that their children could still get malaria. There seemed to be less understanding of the purpose of the trial where there was least amount of CE, and where recruitment was considered by trial staff to be ‘easiest’. That is, the proportion of parents from each site who reported prevention of malaria as the main purpose of the activities was 40.3% (n = 27/67) in site A, 36.4% (n = 12/33) from site B, and 23.0% (n = 23/100) from site C. The differences were statistically significant between sites A and C [x^2^ (1), = 5.72, *P* < 0.017].

After one year of experience of the trial, 93% (n = 186) of parents reported that they were content with their decision to join the trial, and only 1% (n = 2) said that they would not consider joining another future similar trial. This suggests an overwhelming proportion of participating parents did not later regret the decisions they made about their child’s enrolment. Parents’ reasons for their positive perceptions of the trial were: the rumours and concerns circulating early on in the trial had been shown to be untrue; trial participants’ access to free and good quality health care; the perception that the vaccine was successful; and the caring attitude of the trial staff (see also Table 7).

### Continuous engagement and feedback of findings

There were some concerns raised by community gatekeepers and staff that CE activities began to reduce over time, once trial numbers had been reached:

I think there is a gap there because the trend is, once you give this information and maybe … you recruit numbers [required], then the forum for holding barazas [public meetings] and continuing to give information comes to an end, because we’ve got the target (IDI05_male, trial staff).

This gap was discussed with the trial team, and addressed through ensuring that trial or CLG staff regularly attended community leaders’ meetings. In addition, and as planned from the outset, the feedback of preliminary results to the study communities in November 2011 and October 2012 became additional opportunities to engage with participants and community leaders. In each site, feedback meetings were organised for participating families, community leaders and local health facility staff over two days, immediately following the official global launch of results [21]. Thereafter FWs visited each participating household with official letters summarising key meeting information.

Feedback meetings were well attended with over 66% (n = 598/904) of participating households represented in the two-day meetings in 2011. Meetings provided an opportunity to reiterate the study aims and importance of the trial, and to thank participants’ parents for their participation in the trial. The preliminary trial results communicated see also [21], highlighted the trial vaccine continued to be safe and protect against clinical malaria by 55.8% among children aged 5 to 17 months at enrolment, who had received three doses of the vaccine and who were followed up for 12 months. These findings appeared to further alleviate concerns about the trial and encourage participating parents to keep their children in the trial:

… mothers [parents] were very happy because at least they saw that their efforts of coming here [at the health facility] for the vaccine was not wasted and so there was something truly going on. Therefore when they got these results they knew that this study was real. Another thing they were happy about was the way they were being followed up by those [fieldworkers] who oversee the project at their village. It was a clear indication that they [parents] were a very important link in the study (FGD06_male, fieldworker, site A).

## Discussion

The potential importance of CE, but also the complexity and possibility of unexpected negative outcomes, has led to calls for greater documentation of CE processes in practice. In this paper, we have shared experiences of formal CE for a paediatric randomized controlled malaria vaccine trial conducted in three different sites in one geographical area of Kilifi County in Kenya. Having this study conducted alongside and feeding into on-going CE for the trial, and by social scientists (VA, DK, VM, SM) employed by the wider KWTRP, could have reduced our independence and critical analysis. However, we feel we benefited from both an insider status (the wider KWTRP) and outsider status (from the trial outcome); including building trust with the trial team, capturing the shifting day-to-day trial experiences, and ensuring that concerns we identified were discussed and - where appropriate - acted upon.

Overall, the CE approach and activities were considered appropriate by the various stakeholders interviewed (trial researchers, FWs, community leaders and parents), with activities apparently assisting in reducing concerns and misconceptions among potential participants, and increasing visibility, awareness of and trust in trial staff. However, several findings warrant further discussion, including if and how rumours and concerns about the trial and KWTRP were fuelled by information sheets and consent forms, the apparently low levels of trial understanding despite the amount of CE, and what emerge as the contrasting and sometimes conflicting goals of different actors involved in CE.

### Consent information feeding into rumours and concerns in local communities

One goal of CE among researchers was to build levels of trust so that potential participants would consider their child’s participation; a more specific goal was to ensure that participants’ parents were adequately informed about the study, and able to make their own decision about participation without undue influence by others.

In this trial we document very similar rumours and concerns to those we have reported elsewhere on the Kenyan Coast [13-15] and that have been identified across Africa [25-27]. As many of these papers argue, these rumours should be taken seriously as a real concern, and as an expression of uncertainty, tension and ethical comment in settings with long histories of poverty, inequity and invasion. The strength for communities of rumours lies in the importance but difficulty of confronting them: simply re-stating the scientific facts or supplementing explanations with demonstrations is inadequate [13]. The CE activities for the trial discussed in this paper were therefore only partially about promoting understanding of study details; also crucial was reducing suspicion and fears, exhibiting behaviours such as truthfulness, concern, and fairness, and ultimately building trust.

We have shown how information in a consent form (and broader CE and consent processes) can not only fail to clear anxieties and fears, but even fuel them. Information can be interpreted very differently by trial staff, reviewers of consent forms, and community members: messages designed to clarify issues and rights, even when designed following carefully developed local processes [28], can be drawn upon by community members to raise concerns and challenge researchers in powerful ways. For example in this study, anxieties raised with information on risk and confidentiality in information sheets led to shifts in the foci and tone of CE activities. Although rewording of forms may have reduced some of the concerns, the specific words triggered and enabled discussions about pre-existing tensions and worries that required positive reaction and engagement by the research team to build relationships and trust [13,14]. These findings also illustrate the very different perceptions there are of (signed) consent forms and processes in many non-Western settings to the Western countries in which they originate [29], and how they potentially feed into very different concepts of study-related risk that different actors might bring to an engagement activity [30].

### Community engagement and levels of understanding of the trial

Having noted that CE activities were as much about relationship building as information sharing, clearly the two are inter-related, and a key aim of CE here as elsewhere is to support consent processes. We recorded low levels of understanding of key trial information one year after enrolment, but significantly higher levels of understanding in the first site where there was greatest CE. While there might be some temptation to attribute greater understanding to higher levels or different forms of CE, these differences could have resulted from other factors such as amount of previous exposure to research (site C had greater exposure potentially leading to less confusion with other studies).

These data on understanding should be interpreted with caution. Firstly, ‘understanding’ is notoriously difficult to define and measure, particularly through a relatively simple survey tool conducted long after the consenting process [31]. A particular challenge in resource poor settings is the possible crowding out of research information over time by more prominent interests and concerns among participants or their carers, such as access to study benefits including much needed free medical care [6,12,32]; certainly a strong possibility in this trial. Secondly, and in part because of the challenges of measuring understanding and the debates about what to do in the case of apparent ‘inadequate’ levels of understanding among potential participants, some authors have argued that the key concern in consent processes should be that people are *empowered* to make decisions that are appropriate for themselves about participation [31]; decisions that they do not go on to later regret once they experience what the study actually involves (as opposed to hearing about it before it begins). Our data suggested that participants’ parents generally did not regret participating one year into the trial, although it is important that this does not equate to their having made an informed decision about participation at the outset, or about staying in the trial. Some parents who did not consent apparently later regretted that decision (anxieties about risks such as children dying or health deteriorating subsided, and participating children enjoyed study benefits), but it is difficult to imagine how this situation might have been prevented beyond the activities described above.

The apparently low levels of understanding one year into the trial remains a concern. Although this is far from unique to this trial or setting [5], it illustrates the very different priorities and concerns different actors bring to a research encounter, and the importance of continuing CE over time. For this trial, feedback activities provided an opportunity for key messages about the trial to be reiterated and discussed. This appeared to be appreciated and to improve understanding, but this understanding needs to be documented more quantitatively.

### Complex and contrasting goals for CE among key actors

Over the course of the observations and interviews it became clear that the trial team and others involved with CE activities had different motivations or goals for involvement in the trial. These goals were not articulated but revealed through actors’ additional and un-anticipated activities (see Table 6 for an illustrative extract), which reveal the very different realities and interests brought to CE activities and interactions. CE can therefore be understood as interface activities; where different interests, relationships, modes of rationality and power intersect, and sometimes conflict [33]. CE activities can therefore take on a life of their own; transforming planned interventions into socially constructed and (re) negotiated processes.

The translation and constant reshaping of activities involves diverse and multi-layered practices of power. In some cases the most commonly recognised form of power - authoritative power - can be used to dominate and prevent others from expressing their opinions and actions. For example, we reported incidences of chiefs and elders trying to exercise their authoritative power over community members who were not keen for their children to participate. These incidents have worrying implications for voluntariness in consent processes, protected against to some extent by final information sharing and consent being conducted individually by trained clinical staff not embedded in study communities. In other cases, those considered to be relatively powerless exert their own influence on activities and interactions through exercising their discretionary power. For example, FWs returned to households to reassure participants where they felt that leaders had been inappropriately persuasive, or community members spread rumours which could be challenging for researchers to respond to.

Beyond the functioning of the trial itself, goals of actors, and how they are responded to, can also have wider implications. Thus for example, and as we have described elsewhere [10], CHWs’ health care roles in the community were under-resourced at the time of this trial, and therefore depended largely on their intrinsic motivation such as their ability to gain social recognition, knowledge and to make a social contribution through performing their roles. These intrinsic motivations may have been crowded-out by the extrinsic motivations introduced by the trial as part of the CE activities.

## Conclusions

The everyday engagement activities of all actors involved in this trial, including the use of authoritative and discretionary power, had the potential to both assist with and undermine the trial team members’ goals for CE. Of interest is that the trial teams’ goals for engagement were relatively clear and openly shared with all from the outset. Other actors’ hopes and expectations were not openly sought and discussed, but emerged over the course of engagements, in some cases in frustrating or worrying ways. Encouraging open discussion of all actors’ intentions and goals from the outset takes time, risks raising expectations that cannot be met, and is complex given the necessarily constantly shifting nature of who is engaged with over time and in what way. Counter arguments might be that some of the unanticipated activities that had the potential to undermine the trial team members’ intentions for CE might have been avoided if different interests had been more fully understood by all from the outset.

## Abbreviations

CABs: Community Advisory Boards; CAGs: Community Advisory Groups; CAST: Community Advisory for Study Teams; CBO: Community Based Organisation; CE: community engagement; CHWs: community health workers; CLG: Community Liaison Group; DHC: dispensary health committee; DK: do not know; FGD: focus group discussion; FWs: field workers; HF: health facility; HSR: Health Systems Research department; ICF: informed consent form; IDI: in-depth interview; KEMRI/WTRP: Kenya Medical Research Institute/Wellcome Trust Research Programme; KHDSS: Kilifi Health and Demographic Surveillance System; MoH: Ministry of Health.

## Competing interests

The authors declare they have no competing interest.

## Authors’ contributions

VA assisted in study design, tool development, data collection and analysis, and in drafting the manuscript. DK assisted with study conception and design, as well as data collection, analysis and manuscript revisions. DM and BK were involved in design and implementation of the trial’s CE activities and manuscript revision. VM, PN and SM were involved in study conception and design, analysis, interpretation of data, manuscript preparation and revision. In addition, SM was the overall project supervisor. All authors read and approved the final manuscript.
